# YTH Domain: A Family of *N*^6^-methyladenosine (m^6^A) Readers

**DOI:** 10.1016/j.gpb.2018.04.002

**Published:** 2018-04-30

**Authors:** Shanhui Liao, Hongbin Sun, Chao Xu

**Affiliations:** 1 Heifei National Laboratory for Physical Sciences at the Microscale and School of Life Sciences, University of Science and Technology of China, Hefei 230027, China; 2 School of Food and Biological Engineering, Zhengzhou University of Light Industry, Zhengzhou 450002, China; 3 CAS Key Laboratory of Structural Biology, University of Science and Technology of China, Hefei 230027, China

**Keywords:** RNA modification, RNA methylation, RNA demethylation, YT521-B homology, Epitranscriptome

## Abstract

Like protein and DNA, different types of RNA molecules undergo various modifications. Accumulating evidence suggests that these **RNA modifications** serve as sophisticated codes to mediate RNA behaviors and many important biological functions. *N*^6^-methyladenosine (m^6^A) is the most abundant internal RNA modification found in a variety of eukaryotic RNAs, including but not limited to mRNAs, tRNAs, rRNAs, and long non-coding RNAs (lncRNAs)*.* In mammalian cells, m^6^A can be incorporated by a methyltransferase complex and removed by demethylases, which ensures that the m^6^A modification is reversible and dynamic. Moreover, m^6^A is recognized by the **YT521-B homology** (YTH) domain-containing proteins, which subsequently direct different complexes to regulate RNA signaling pathways, such as RNA metabolism, RNA splicing, RNA folding, and protein translation*.* Herein, we summarize the recent progresses made in understanding the molecular mechanisms underlying the m^6^A recognition by YTH domain-containing proteins, which would shed new light on m^6^A-specific recognition and provide clues to the future identification of reader proteins of many other RNA modifications.

## Introduction

The central dogma explains how genetic information is transferred from DNA to RNA to protein [1]. It is well known that epigenetic marks on the nucleosome, including histone modifications and DNA methylation (5-methylcytosine), play important roles in gene regulation by mediating gene transcription events [2], [3]. In addition to DNA and protein, RNA molecules can also be modified. Up till now, more than 100 modifications have been identified in different types of eukaryotic RNAs, including mRNAs, tRNAs, and non-coding RNAs (ncRNAs) [4]. In contrast to the well-studied epigenetic marks, the exact biological roles of most of the identified RNA modifications are largely unknown.

N^6^-methyladenosine (m^6^A), which was discovered in a wide range of cellular RNAs in 1970s [5], [6], [7], is the most prevalent internal RNA modification present in a GAC or AAC motif within almost all types of eukaryotic RNAs examined [8] as well as viral RNAs [9], [10], [11], [12], [13], [14]. On average, there are 3–5 m^6^A sites in each mRNA molecule [15]. m^6^A has been attracting considerable attention because of its important roles in gene regulation [16], genome stability maintenance [17], as well as cell renewal and differentiation [18]. Recent advancements in crosslinking and immunoprecipitation (CLIP) technologies have made it possible to accurately locate this specific mark in cellular RNAs [19].

Similar to other epigenetic modifications, m^6^A is dynamic and reversible, established mainly by the METTL3–METTL14 methyltransferase complex [20], [21] and removed by demethylases including the fat mass and obesity-associated protein (FTO) [22] and AlkB homolog 5 RNA demethylase (ALKBH5) [23]. Although both METTL3 and METTL14 adopt a canonical fold similar to that of other methyltransferases [20], only METTL3 can bind to the methyl donor *S*-adenosyl methionine (SAM or AdoMet), whereas METTL14 acts to modulate the activity of METTL3 and binds to the RNA substrate instead [20], [24], [25]. FTO and ALKBH5 are the only two known m^6^A demethylases found in humans and they both belong to the α-ketoglutarate-dependent dioxygenase family [23], [26]. Interestingly, both FTO and ALKBH5 discriminate single-stranded RNA (ssRNA) from double-stranded RNA (dsRNA), by a unique insertion in the case of FTO and by a loop rigidified by the disulfide bond in the case of ALKBH5 [27], [28], [29], [30]. In addition, ALKBH5 displays comparable activities toward m^6^A-modified ssRNA and *N*^6^-methyldeoxyadenosine (6mA)-modified ssDNA [30]. Although the *in vivo* biological relevance of 6 mA ssDNA demethylation by ALKBH5 remains unknown, 6 mA has been identified in eukaryotic genomes by several groups [31], [32], [33].

The regulatory role of m^6^A on RNA molecules is similar to that of epigenetic marks on chromatin [34], which could be achieved in two ways, *i.e.*, *cis* and *trans*. In the *cis* mode, the effect of m^6^A on the RNA structure is similar to that of epigenetic marks on the nucleosome. Incorporation of the methyl moiety at the *N*^6^ atom of adenosine renders the m^6^A–U pair energetically unfavorable [35], which may cause destruction of the stem loop where it resides and lead to further global conformational rearrangement of the RNA [8]. In addition, m^6^A can mediate RNA functions in a *trans* mode through the recruitment of specific proteins or protein complexes [8].

**Figure 1:**
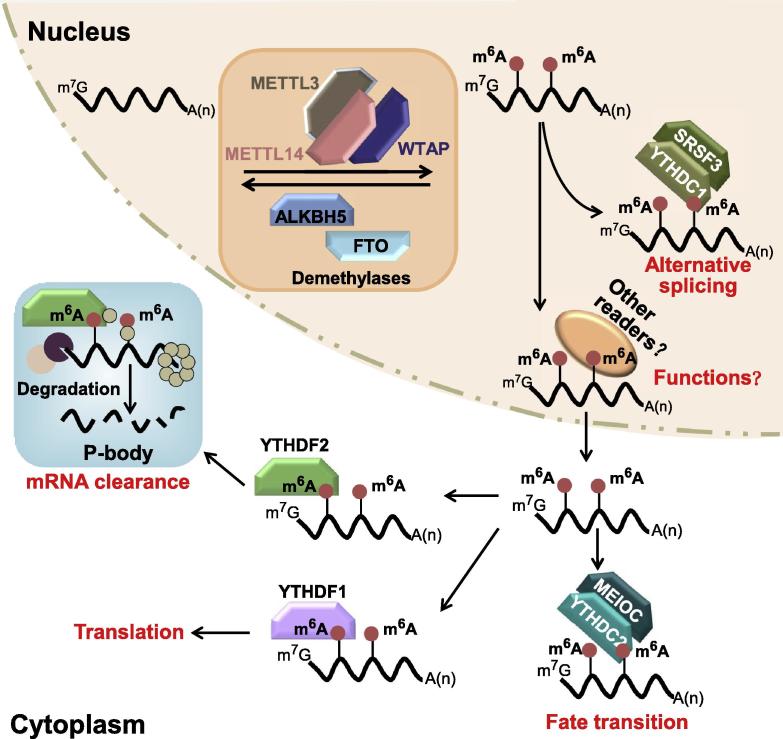
**Regulatory roles of m^6^A effector proteins** The m^6^A effectors include the writer protein (m^6^A methyltransferase complex; METTL3–METTL14), eraser proteins (RNA demethylases; FTO and ALKBH5), and reader proteins (YTHDC1, YTHDC2, YTHDF1, YTHDF2). Effectors of m^6^A are labeled to indicate their roles in mediating the functions of RNA molecules: splicing, translation, stability, localization, *etc.* ALKBH5, AlkB homolog 5 RNA demethylase; FTO, fat mass and obesity-associated protein; MEIOC, meiosis-specific coiled-coil domain-containing protein; METTL3, methyltransferase like 3; P-body, processing body; SRSF3, serine/arginine-rich splicing factor 3; WTAP, Wilms’ tumor 1 associated protein; YTHDC1, YTH domain containing 1; YTHDF1, YTH domain family, member 1.

The YT521-B homology (YTH) domain serves as the module for recognizing m^6^A in a methylation-dependent manner [36], [37], [38]. There are five YTH domain-containing proteins in humans, namely, YTHDC1, YTHDC2, YTHDF1, YTHDF2, and YTHDF3. YTHDF2 is the first protein, of which the m^6^A-associated function has been well studied [33]. After being targeted to a specific site via m^6^A recognition, YTHDF2 recruits the CCR4-NOT deadenylase complex to destabilize and further decay target mRNAs (Figure 1) [37], [39]. Binding of YTHDF1 to m^6^A-modified mRNA increases the translation efficiency of the mRNA independent of the m^7^G cap (Figure 1) [40]. YTHDC1, the only known m^6^A reader in the nucleus, has been reported to be involved in exon selection during gene splicing (Figure 1) [17]. YTHDC2 is a putative RNA helicase [41], [42] that forms a complex with the meiosis-specific coiled-coil domain-containing protein (MEIOC) to regulate RNA levels during meiosis through recognizing m^6^A by its YTH domain (Figure 1) [42]. Thus, by targeting different complexes to specific sites via direct binding to m^6^A, the YTH domain-containing proteins participate extensively in *post*-*transcriptional regulation* by regulating splicing, translation, localization, and lifetime of RNAs (Figure 1) [43]. By reading and interpreting the m^6^A mark, these proteins play important roles in gene regulation, DNA repair, and cell fate determination [44].

The specific m^6^A recognition mode by the YTH domain had remained largely unknown until the structure of the first human YTH complex, the YTHDC1 YTH domain with the 5-mer GG(m^6^A)CU RNA, was solved in 2014 [38]. Immediately thereafter, several structures of human YTH domain-containing proteins have also been reported, including the YTH domains of YTHDF1 and YTHDF2 with their respective m^6^A-modified RNA ligands, the structure of the YTHDF2 YTH domain alone, and one nuclear magnetic resonance structure of the YTHDC1 complex [45], [46], [47], [48]. Besides the YTH family proteins, other RNA-binding proteins (RBPs), such as heterogeneous nuclear ribonucleoproteins A2/B1 (HNRNPA2B1) [49], embryonic lethal, abnormal vision-like protein 1 (ELAVL1) [50], and insulin-like growth factor 2 mRNA-binding proteins 1–3 (IGF2BP1–3) [51], are also suggested to be potential m^6^A-binding proteins, albeit awaiting further confirmation. We aim to summarize the progresses made in unraveling the structural features of the YTH family proteins, including the m^6^A-binding specificity and sequence selectivity. Furthermore, we also provide mechanistic insights into the search for new m^6^A reader proteins based on known rules of m^6^A recognition.

## Human YTH domain-containing proteins

**Figure 2:**
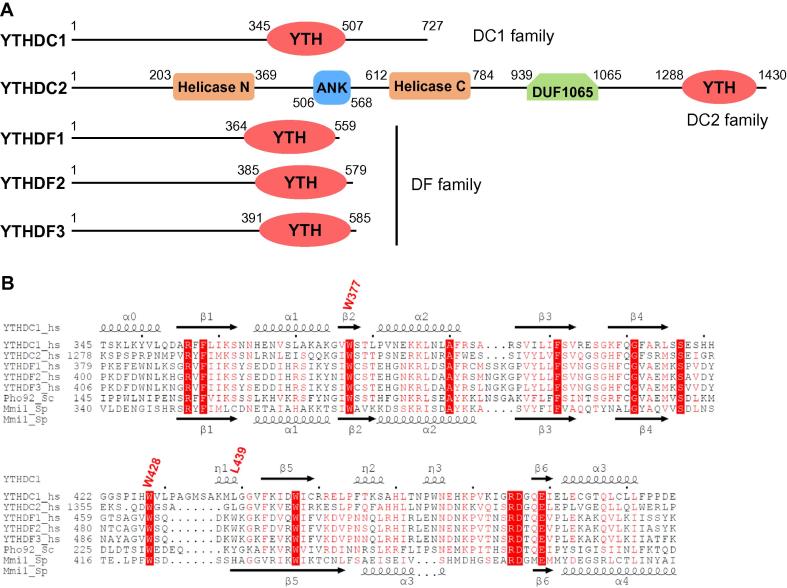
**Domain organization of human YTH domain-containing proteins and sequence alignment of human and yeast YTH domains** **A.** Domain architecture of human YTH domain-containing proteins: YTHDC1 (UniProt ID: Q96MU7), YTHDC2 (UniProt ID: Q9H6S0), YTHDF1 (UniProt ID: Q9BYJ9), YTHDF2 (UniProt ID: Q9Y5A9), and YTHDF3 (UniProt ID: Q7Z739). **B.** Sequence alignment of the YTH domains of human YTHDC1, YTHDC2, YTHDF1–3, budding yeast Pho92 (UniProt ID: Q06390), and fission yeast Mmi1 (UniProt ID: O74958). Key hydrophobic residues of YTHDC1 forming the m^6^A-binding pocket (W377, W428, and L439) are indicated in red. YTH, YTH domain; ANK, ankyrin repeats; helicase N, helicase N-terminal domain; helicase C, helicase C-terminal domain. YTHDF1, YTHDF2, and YTHDF2 are similar to each other, whereas YTHDC1 and YTHDC2 are considered as different subtypes.

The YTH domain is present in 174 different proteins and is evolutionarily conserved across the eukaryotic species [52]. Early functional studies of YTH domain-containing proteins, such as YT521-B [53] and Mmi1 [54], [55], have implied their potential roles in RNA metabolism. Although YTH domain-containing proteins are putative RBPs, their exact binding ligands had remained unknown until two reports discovered that mammalian YTH family members are the candidates of m^6^A readers [36], [37]. By searching through the human genome, five YTH domain-containing proteins are found, namely, YTHDF1–3 and YTHDC1–2, all of which are conserved in mammalian genomes (Figure 2A). On the basis of their primary sequences and domain organizations, these five human YTH domain-containing proteins can be classified into three categories: YTHDC1 (DC1 family), YTHDC2 (DC2 family), and YTHDF1–3 (DF family) (Figure 2A). YTHDC1 is a nuclear protein involved in gene splicing, whereas YTHDF1–3 are cytoplasmic m^6^A readers [37]. YTHDC2 is a putative RNA helicase that, aside from the YTH domain, contains the helicase domain, ankyrin repeats, and DUF1065 domain (Figure 2A), which may act as a scaffold molecule in regulating spermatogenesis [41].

## Structural features of the YTH complexes

**Figure 3:**
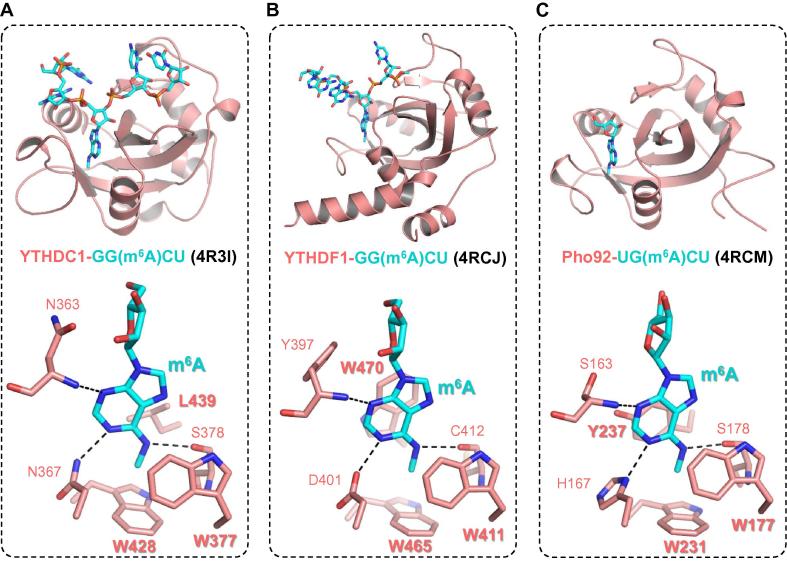
**Structural analysis of YTH domain complexes with m^6^A-containing RNA molecules** **A.** Structure of the YTHDC1-GG(m^6^A)CU complex (PDB ID: 4R3I). **B.** Structure of the YTHDF1-GG(m^6^A)CU complex (PDB ID: 4RCJ). **C.** Structure of the Pho92-UG(m^6^A)CU complex (PDB ID: 4RCM). Proteins are shown in red cartoon, with m^6^A-binding residues shown as red sticks. RNA molecules are shown as cyan sticks. The hydrogen bonds between the protein and m^6^A are indicated using black dashed lines.

Although all five human YTH domain-containing proteins share a homologous YTH domain, the biological functions of these proteins remain unknown until YTHDF2 is reported to affect the lifetimes of mammalian mRNAs through recognizing m^6^A by its YTH domain [37], indicating that the YTH domain serves as the m^6^A binding module. Subsequent determination of the crystal structures of the YTHDC1 YTH domain, alone and together with GG(m^6^A)CU RNA, helps unravel the mechanisms underlying m^6^A recognition and sequence selectivity [38]. The YTH domains share a conserved α/β fold (Figure 2B), which consists of four or five α helices and six β strands [38]. These six β strands form a β barrel, with the α helices packed against the β strands to stabilize the hydrophobic core (Figure 3) [38].

In the YTHDC1–m^6^A complex, the RNA molecule lies in the positively-charged groove of the protein, with m^6^A buried in a deep cleft formed by three hydrophobic residues, W377, W428, and L439 (Figure 3A) [38]. Specifically, the methyl–π interactions between the methyl group of m^6^A and the rings of the two tryptophan residues constitute the basis of m^6^A-specific recognition, consistent with the fact that the YTHDC1 YTH domain exhibits binding affinity toward m^6^A-modified RNAs, but not unmodified RNAs [38]. The m^6^A binding mode of the YTH domain is somewhat similar to that of the methyllysine recognition by Royal family domains, which also utilize an aromatic cage pocket to accommodate the methyllysine residue [56]. In addition to the methylation-dependent interactions, m^6^A also forms base-specific hydrogen bonds with N363, N367, and S378 of YTHDC1 (Figure 3A) [38]. Of note, the m^6^A-binding pocket of YTHDC1 can accommodate m^6^A, but not *N*^6^,*N*^6^-dimethyladenosine (m^6,6^A), since introducing another methyl group at *N*^6^ would not only disrupt the hydrogen bond between S378 and *N*^6^ of m^6^A but also cause steric clash with the backbone of S378 (Figure 3A). Besides m^6^A-specific binding, electrostatic interactions between YTHDC1 and the RNA molecule also contribute to formation of the complex, such as the hydrogen bond between the guanosine at −2 position (G−2) and D476 of YTHDC1, cation–π interaction between the cytosine following m^6^A (C+1) and R475 of YTHDC1, as well as several hydrogen bonds between YTHDC1 and sugar-phosphate backbone atoms of RNA [38].

With the elucidation of the YTHDC1–m^6^A complex, two other complexes, the YTH domains of YTHDF1 and YTHDF2 with their respective m^6^A-modified RNA ligands, have also been reported [45], [46]. In both complexes, m^6^A is recognized in a manner similar to that observed in the YTHDC1–m^6^A complex (Figure 3B). m^6^A is positioned into a positively-charged pocket of YTHDF1, formed by the side chains of W411, W465, and W470. The methyl group of m^6^A points to the ring of W465 and is positioned between the rings of W411 and W470 [46]. The methyl–π interactions between m^6^A and the three tryptophan residues constitute the methylation-dependent recognition mode. Furthermore, the YTH domain of yeast Pho92, the only YTH domain-containing protein in *Saccharomyces cerevisiae*, adopts the canonical YTH fold and possesses the m^6^A-binding pocket (Figure 3C) [46], which is formed by W177, W231, and Y237, suggesting a conserved m^6^A recognition mode in eukaryotes (Figure 3C). Of note, in all of these m^6^A-binding pockets, the residues W411 and W465 of YTHDC1 are absolutely conserved in all human YTH domains, whereas the third residue could be tryptophan, tyrosine, or leucine (Figure 2B), indicating that these YTH domains described above not only adopt a common architecture but also share a conserved m^6^A-binding pocket.

Comparison of the binding affinity between YTHDC1 and m^6^A with that between YTHDF1 and m^6^A shows that the YTH domain of YTHDC1 binds to the 5-mer m^6^A-modified RNA ∼10 folds more strongly than does that of YTHDF1. Detailed structural analysis indicates that YTHDC1 utilizes N367 to form a hydrogen bond with *N*^1^ of m^6^A, whereas the corresponding residue in YTHDF1 is D401 (Figure 3A and B). Under neutral or basic pH conditions, *N*^1^ of m^6^A cannot serve as the hydrogen donor to form one hydrogen bond with an aspartic acid residue; instead, it serves as the hydrogen acceptor to be hydrogen bonded to an asparagine, such as N367 of YTHDC1. Only under acidic pH conditions, the protonation of *N*^1^ might make it possible for m^6^A to form a hydrogen bond with D401 of YTHDF1 (Figure 3B). Further work is required to investigate the pH-dependent interactions between YTHDF1 and m^6^A-modified RNA, which might explain the apparently weak binding of YTHDF1 to short m^6^A-modified RNAs.

**Figure 4:**
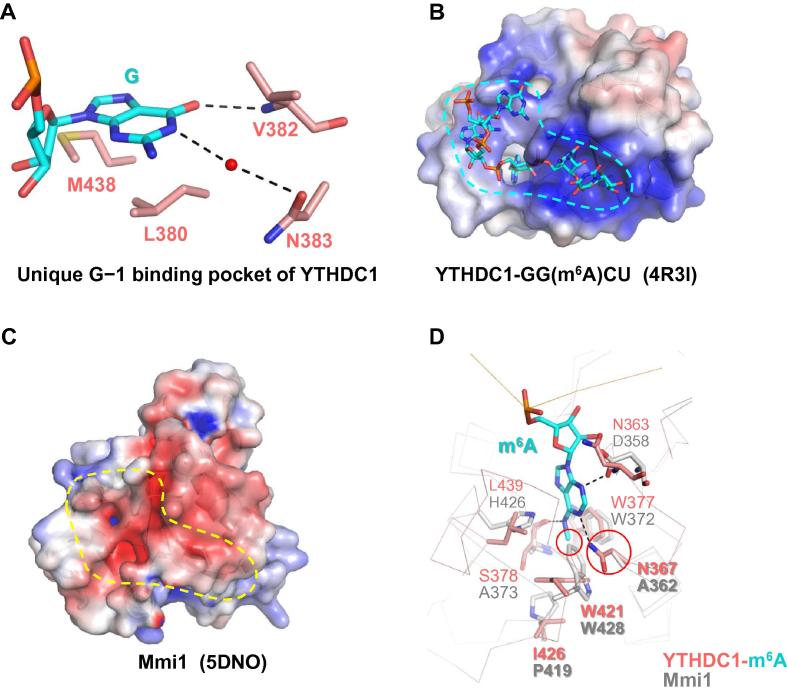
**Structural comparison of YTHDC1 and Mmi1** **A.** G−1 binding pocket of YTHDC1. Residues involved in recognizing G−1 are shown as red sticks and G−1 is shown as cyan sticks. The water molecule is shown as a red sphere, and hydrogen bonds are indicated using black dashed lines. **B.** Electrostatic potential surface of YTHDC1–GG(m^6^A)CU, with the RNA-binding groove of the YTH domain in YTHDC1 highlighted (PDB ID: 4R3I). **C.** Electrostatic potential surface of the corresponding surface of Mmi1 (PDB ID: 5DNO), with the corresponding region of Mmi1 highlighted, indicating that this region is not favorable for binding RNA. **D.** Superimposed structures of YTHDC1 (red) and Mmi1 (gray) on the m^6^A-binding pocket of YTHDC1. The m^6^A-binding residues of YTHDC1, as well as the corresponding residues of Mmi1, are shown as sticks. m^6^A is shown as cyan sticks.

Despite the common m^6^A-binding pocket, the YTH domains display different binding preferences. The YTH domain of YTHDC1 prefers a guanosine residue at a position preceding m^6^A (G−1), as confirmed by binding experiments using both photoactivatable-ribonucleoside-enhanced CLIP (PAR-CLIP) and isothermal titration calorimetry (ITC) [36], [38]. In the YTHDC1–m^6^A complex, the G−1 residue stacks with L380 and M438 of YTHDC1, and forms hydrogen bonds with V382 and N383 (Figure 4**A**) [38]. G-to-A substitution at the −1 position would disrupt the base-specific hydrogen bonds and lead to steric clash between A−1 and the backbone of V382; therefore, adenosine at the −1 position is not favored [38]. Collectively, hydrophobic interactions and hydrogen bonds confer the binding preference for guanosine at the −1 position on YTHDC1 [38]. Other human YTH domains do not seem to contain the G−1 binding pocket, and neither does the YTH domain of yeast Pho92, indicating that the Pho92 YTH domain might be evolutionarily more similar to that of YTHDF1 than to that of YTHDC1. Furthermore, the difference in sequence selectivity between the YTH domain of YTHDC1 and other human YTH domains may reflect the differential m^6^A-binding demands in nucleus and cytoplasm [38], [46].

## The pocket of YTH domain governs the m^6^A-specific recognition

In contrast to Pho92, Mmi1 from fission yeast contains a YTH domain that does not exhibit m^6^A-specific binding toward RNA ligands, although the Mmi1 YTH domain also adopts the canonical YTH fold [57]. Structural comparison of the YTH domains of YTHDC1 and Mmi1 indicates that while YTHDC1 contains a large positively-charged groove to position the m^6^A-modified RNA (Figure 4B), the corresponding surface of Mmi1 is negatively-charged, which impairs the binding of its YTH domain to negatively-charged RNA backbones (Figure 4C). In addition, when superimposing the m^6^A-binding pocket of YTHDC1 with that of Mmi1, it is found that the key m^6^A-binding residues of YTHDC1 are not conserved in Mmi1, with N367 of YTHDC1 replaced by an alanine residue in Mmi1, which would disrupt the base-specific hydrogen bond (Figure 4C). Moreover, W428 of Mmi1 rotates its ring plane by ∼90° to avoid potential clash with the Mmi1 P419, which completely blocks the entry of the m^6^A-modified nucleotide into this pocket (Figure 4D).

Mmi1 can bind to an unmodified RNA motif named as the DSR core motif (5′-U(U/C)AAAC-3′) [57]. Recent structural studies of the Mmi1 YTH domain in complex with the DSR motif have revealed that Mmi1 binds to the RNA via a positively-charged groove formed by its α 4 helix, as well as β 3 and β 4 strands, which is distinct from the YTHDC1–m^6^A surface [57], [58]. Of note, the methylation of adenosine within the DSR motif would weaken rather than enhance the binding [57]. The diversity in the RNA-binding ability of the two yeast YTH domains implies that they are deviated from each other during evolution, albeit without altering the fold.

**Figure 5:**
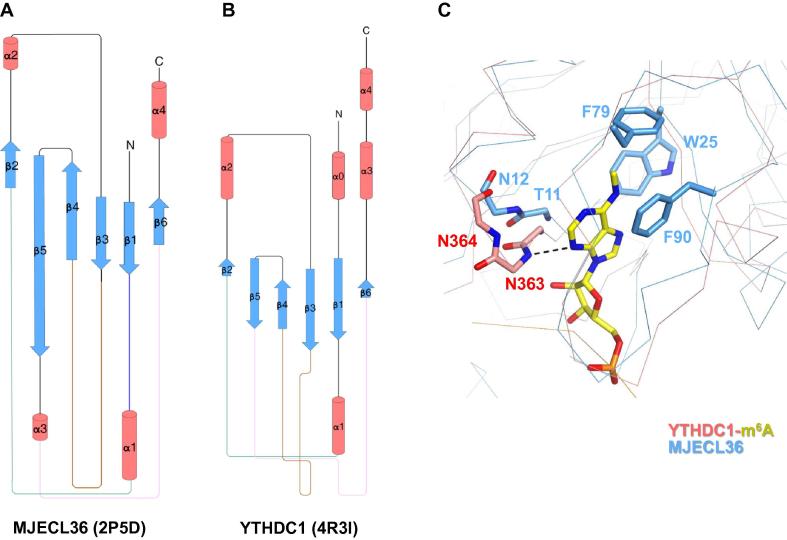
**Topologies of the YTHDC1 YTH domain and MJECL36** **A.** Topology of the YTH domain of human YTHDC1 (PDB ID: 4R3I). **B.** Topology of MJECL36 (PDB ID: 2P5D). α helices and β strands are indicated by red cylinders and blue rectangle arrows, respectively. **C.** Superposition of the structures of MJECL36 (blue) and the YTHDC1 (red)-m^6^A (yellow) complex, with the two proteins shown as ribbons. The aromatic residues of MJECL36, as well as the m^6^A-modified nucleotide, are shown as sticks. The backbones of T11 and N12 of MJECL36, as well as of N363 and N364 of YTHDC1, are also shown as sticks to indicate their different conformations. Unlike N363 of YTHDC, T11 of MJECL36 could not form the backbone hydrogen bond with *N*^3^ of m^6^A. The hydrogen bond is indicated using a black dashed line.

A search within the Dali database reveals other proteins containing domains with an architecture similar to that of the YTH domain, such as MJECL36 of *Methanocaldococcus jannaschii* (PDB ID: 2P5D; Figure 5A and B). Similar to that of the YTHDC1 YTH domain, the fold of MJECL36 consist of β strands arranged in an order of 2-5-4-3-1-6 (Figure 5A and B). In MJECL36, an aromatic cage is formed by the rings of W25, F79, and F90, which could be superimposed with the aromatic cage of YTHDC1, and accommodates the m^6^A-modified nucleotide (Figure 5C). However, residues at the C-terminal end of β1 in MJECL36, T11 and N12, deviate from those of YTHDC1, N363 and N364, potentially disrupting the backbone hydrogen bond formed between *N*^3^ of m^6^A and N363 of YTHDC1 (Figure 5C). Consistently, MJECL36, albeit with a YTH-like fold, does not exhibit any detectable m^6^A-binding affinity [46]. It seems that the size of the pocket, as well as the aromatic residues that reside therein, confers the m^6^A-binding ability on the YTH domain. The similar topology and distinctive functions between the YTH domain and MJECL36 indicate that they may have originated from a common ancestor, but the YTH domain in higher eukaryotes probably has acquired adaptive functions after a long period of evolution.

## Other potential m^6^A binders

Besides the YTH domain-containing proteins, eukaryotic initiation factor 3 (eIF3) has also been reported as an m^6^A reader [59]. The cap-binding protein eIF4 is essential for translation initiation [59]. However, eIF4-independent translation initiation can occur in case of eIF4 loss of function or viral mRNA translation [60]. Meyer et al. [59] report that eIF3 facilitates eIF4-independent translation of mRNAs depending on the m^6^A modification in their 5′ UTRs.

HNRNPA2B1, a RBP that contains the RNA recognition motif (RRM) domain, has also been reported as an m^6^A reader. Alarcón et al. find that HNRNPA2B1 binds to m^6^A-rich sites in the transcriptome [49]. However, a recent study on the complex structure of HNRNPA2B1 with RNA shows that no aromatic pocket is found in the RRM domain of HNRNPA2B1, which prefers unmodified RNA ligands [61]. Therefore, HNRNPA2B1 might not bind to m^6^A directly, although we could not rule out the possibility that the binding occurs via other effectors. Another RRM domain-containing protein, embryonic lethal, abnormal vision-like protein 1 (ELAVL1), can be pulled down by m^6^A-containing RNAs. ELAVL1 contains three RRM domains, all of which are homologous to the determined RRM structures and do not seem to contain the m^6^A-binding pocket [50]. Although we could not exclude the possibility that ELAVL1 might recognize m^6^A via other regions, it is also possible that ELAVL1 binds to other sequences rather than the m^6^A site itself, as implicated by the difference between the sequence of the m^6^A site and the ELAVL1-binding motif [50].

Very recently, IGF2BPs are reported to enhance mRNA stability and mediate translation in an m^6^A-dependent manner [51]. IGF2BPs contain tandem KH domains and KH domain is a conserved ssRNA-binding domain that usually appears as tandem repeats in proteins [62]. Whether the tandem KH domains of IGF2BPs serve as the reader of m^6^A requires further investigation. One possibility is that some intrinsically-disordered regions flanking the KH domains may endure conformational conversion to enable the m^6^A binding by providing additional contacts. For example, although the RGG motif from the human fragile X mental retardation protein (FMRP) alone is disordered, it becomes ordered after binding to the major groove of the G-rich RNA duplex–quadruplex junction [63].

## Concluding remarks and outlook

In the past decades, the roles of histone modifications and DNA methylation have been well studied. In contrast, although >100 RNA modifications have been discovered *in vivo*, their exact roles remain elusive. As the hallmark of RNA epigenetics, m^6^A mediates the functions of eukaryotic RNAs extensively [64]. The YTH domain represents a family that recognizes the m^6^A mark directly. By recruiting different complexes to target m^6^A sites, the YTH domain-containing proteins, as well as other potential m^6^A-binding proteins, contribute to gene regulation post-transcriptionally in many aspects, such as splicing, translation, localization, and lifetime.

Despite the progress made in understanding the m^6^A effectors in the past few years, some questions remain to be answered. Are there more proteins that recognize m^6^A directly? How should we go about discovering the reader proteins of many other RNA modifications, such as the *N*^1^-methyladenosine (m^1^A) [65], 5-methylcytosine (m^5^C) [66], *N*^6^,2′-*O*-dimethyladenosine (m^6^Am) [67], and pseudouridine (ψ) [68]? Could a better understanding of m^6^A-binding proteins facilitate our search for readers of other RNA modifications? Is it possible that the YTH domain serves as the readers of other modified RNAs?

Detailed structural analysis has revealed that the m^6^A base fits into the YTH domain pocket and forms several base-specific hydrogen bonds with the YTH domain residues. Therefore, it is unlikely that the same pocket of the YTH domain could recognize other modified bases other than m^6^A. Even for m^6^Am, introducing a methyl moiety would disrupt the hydrogen bond between the C2′-ribosyl hydroxyl oxygen and the side chain of N363 in YTHDC1. It is possible that YTHDC1 could accommodate m^6^Am by changing the conformation of N363. Whether the YTH domain-containing proteins possess the m^6^Am-binding ability requires further examinations both *in vitro* and *in vivo*.

In the epigenetic field, the structural information of known mediators or readers of histone acetyllysine and methyllysine has been used to guide the design and development of chemical probes [69]. Interestingly, some of the small molecules designed serve as inhibitors of protein–protein interactions rather than inhibitors of enzymes [70]. Considering the similar characteristics between the methyllysine-binding pocket and the m^6^A-binding pocket [71], we believe that it is plausible to design small molecules to modulate the functions of RNAs through disrupting the m^6^A recognition by YTH domains. Some human YTH domains have been associated with human diseases, such as cancer or viral *infection*. For instance, YTHDC1 is associated with endometrial cancer [38], while YTHDF1–3 can recognize m^6^A in RNA of human *immunodeficiency virus* 1 (HIV-1) and suppress HIV infection [12]. The structural studies on human YTH domains and other identified m^6^A binders, should help to address the unanswered questions and provide insights into the development of chemical probes and future drug therapies.

## Competing interests

The authors have declared no competing interests.

## References

[1] Crick F. (1970). Central dogma of molecular biology. Nature.

[2] Bannister A.J., Kouzarides T. (2011). Regulation of chromatin by histone modifications. Cell Res.

[3] Goldberg A.D., Allis C.D., Bernstein E. (2007). Epigenetics: a landscape takes shape. Cell.

[4] Boccaletto P., Machnicka M.A., Purta E., Piatkowski P., Baginski B., Wirecki T.K. (2018). MODOMICS: a database of RNA modification pathways. 2017 update. Nucleic Acids Res.

[5] Wei C.M., Moss B. (1977). Nucleotide sequences at the *N*^6^-methyladenosine sites of HeLa cell messenger ribonucleic acid. Biochemistry.

[6] Adams J.M., Cory S. (1975). Modified nucleosides and bizarre 5′-termini in mouse myeloma mRNA. Nature.

[7] Krug R.M., Morgan M.A., Shatkin A.J. (1976). Influenza viral mRNA contains internal *N*^6^-methyladenosine and 5'-terminal 7-methylguanosine in cap structures. J Virol.

[8] Fu Y., Dominissini D., Rechavi G., He C. (2014). Gene expression regulation mediated through reversible m(6)A RNA methylation. Nat Rev Genet.

[9] Lichinchi G., Gao S., Saletore Y., Gonzalez G.M., Bansal V., Wang Y. (2016). Dynamics of the human and viral m(6)A RNA methylomes during HIV-1 infection of T cells. Nat Microbiol.

[10] Gokhale N.S., McIntyre A.B.R., McFadden M.J., Roder A.E., Kennedy E.M., Gandara J.A. (2016). *N*^6^-methyladenosine in flaviviridae viral RNA genomes regulates infection. Cell Host Microbe.

[11] Lichinchi G., Zhao B.S., Wu Y., Lu Z., Qin Y., He C. (2016). Dynamics of human and viral RNA methylation during ZIKA virus infection. Cell Host Microbe.

[12] Tirumuru N., Zhao B.S., Lu W., Lu Z., He C., Wu L. (2016). *N*(6)-methyladenosine of HIV-1 RNA regulates viral infection and HIV-1 Gag protein expression. Elife.

[13] Kennedy E.M., Bogerd H.P., Kornepati A.V., Kang D., Ghoshal D., Marshall J.B. (2016). Posttranscriptional m(6)A editing of HIV-1 mRNAs enhances viral gene expression. Cell Host Microbe.

[14] Chen K., Zhao B.S., He C. (2016). Nucleic acid modifications in regulation of gene expression. Cell Chem Biol.

[15] Narayan P., Rottman F.M. (1988). An *in vitro* system for accurate methylation of internal adenosine residues in messenger RNA. Science.

[16] Xiao W., Adhikari S., Dahal U., Chen Y.S., Hao Y.J., Sun B.F. (2016). Nuclear m(6)A reader YTHDC1 regulates mRNA splicing. Mol Cell.

[17] Xiang Y., Laurent B., Hsu C.H., Nachtergaele S., Lu Z., Sheng W. (2017). RNA m(6)A methylation regulates the ultraviolet-induced DNA damage response. Nature.

[18] Cui Q., Shi H., Ye P., Li L., Qu Q., Sun G. (2017). m(6)A RNA methylation regulates the self-renewal and tumorigenesis of glioblastoma stem cells. Cell Rep.

[19] Hafner M., Landthaler M., Burger L., Khorshid M., Hausser J., Berninger P. (2010). Transcriptome-wide identification of RNA-binding protein and microRNA target sites by PAR-CLIP. Cell.

[20] Liu J., Yue Y., Han D., Wang X., Fu Y., Zhang L. (2014). A METTL3-METTL14 complex mediates mammalian nuclear RNA *N*^6^-adenosine methylation. Nat Chem Biol.

[21] Ping X.L., Sun B.F., Wang L., Xiao W., Yang X., Wang W.J. (2014). Mammalian WTAP is a regulatory subunit of the RNA *N*^6^-methyladenosine methyltransferase. Cell Res.

[22] Jia G., Fu Y., Zhao X., Dai Q., Zheng G., Yang Y. (2011). *N*^6^-methyladenosine in nuclear RNA is a major substrate of the obesity-associated FTO. Nat Chem Biol.

[23] Zheng G., Dahl J.A., Niu Y., Fedorcsak P., Huang C.M., Li C.J. (2013). ALKBH5 is a mammalian RNA demethylase that impacts RNA metabolism and mouse fertility. Mol Cell.

[24] Wang X., Feng J., Xue Y., Guan Z., Zhang D., Liu Z. (2016). Structural basis of *N*(6)-adenosine methylation by the METTL3-METTL14 complex. Nature.

[25] Wang P., Doxtader K.A., Nam Y. (2016). Structural basis for cooperative function of Mettl3 and Mettl14 methyltransferases. Mol Cell.

[26] Gerken T., Girard C.A., Tung Y.C., Webby C.J., Saudek V., Hewitson K.S. (2007). The obesity-associated FTO gene encodes a 2-oxoglutarate-dependent nucleic acid demethylase. Science.

[27] Han Z., Niu T., Chang J., Lei X., Zhao M., Wang Q. (2010). Crystal structure of the FTO protein reveals basis for its substrate specificity. Nature.

[28] Aik W., Scotti J.S., Choi H., Gong L., Demetriades M., Schofield C.J. (2014). Structure of human RNA *N*(6)-methyladenine demethylase ALKBH5 provides insights into its mechanisms of nucleic acid recognition and demethylation. Nucleic Acids Res.

[29] Chen W., Zhang L., Zheng G., Fu Y., Ji Q., Liu F. (2014). Crystal structure of the RNA demethylase ALKBH5 from zebrafish. FEBS Lett.

[30] Xu C., Liu K., Tempel W., Demetriades M., Aik W., Schofield C.J. (2014). Structures of human ALKBH5 demethylase reveal a unique binding mode for specific single-stranded *N*^6^-methyladenosine RNA demethylation. J Biol Chem.

[31] Zhang G., Huang H., Liu D., Cheng Y., Liu X., Zhang W. (2015). *N*^6^-methyladenine DNA modification in *Drosophila*. Cell.

[32] Greer E.L., Blanco M.A., Gu L., Sendinc E., Liu J., Aristizabal-Corrales D. (2015). DNA methylation on *N*^6^-adenine in *C. elegans*. Cell.

[33] Fu Y., Luo G.Z., Chen K., Deng X., Yu M., Han D. (2015). *N*^6^-methyldeoxyadenosine marks active transcription start sites in *Chlamydomonas*. Cell.

[34] Berger S.L. (2007). The complex language of chromatin regulation during transcription. Nature.

[35] Roost C., Lynch S.R., Batista P.J., Qu K., Chang H.Y., Kool E.T. (2015). Structure and thermodynamics of *N*^6^-methyladenosine in RNA: a spring-loaded base modification. J Am Chem Soc.

[36] Dominissini D., Moshitch-Moshkovitz S., Schwartz S., Salmon-Divon M., Ungar L., Osenberg S. (2012). Topology of the human and mouse m^6^A RNA methylomes revealed by m^6^A-seq. Nature.

[37] Wang X., Lu Z., Gomez A., Hon G.C., Yue Y., Han D. (2014). *N*^6^-methyladenosine-dependent regulation of messenger RNA stability. Nature.

[38] Xu C., Wang X., Liu K., Roundtree I.A., Tempel W., Li Y. (2014). Structural basis for selective binding of m^6^A RNA by the YTHDC1 YTH domain. Nat Chem Biol.

[39] Du H., Zhao Y., He J., Zhang Y., Xi H., Liu M. (2016). YTHDF2 destabilizes m(6)A-containing RNA through direct recruitment of the CCR4-NOT deadenylase complex. Nat Commun.

[40] Wang X., Zhao B.S., Roundtree I.A., Lu Z., Han D., Ma H. (2015). *N*(6)-methyladenosine modulates messenger RNA translation efficiency. Cell.

[41] Hsu P.J., Zhu Y., Ma H., Guo Y., Shi X., Liu Y. (2017). Ythdc2 is an *N*(6)-methyladenosine binding protein that regulates mammalian spermatogenesis. Cell Res.

[42] Jain D., Puno M.R., Meydan C., Lailler N., Mason C.E., Lima C.D. (2018). *ketu* mutant mice uncover an essential meiotic function for the ancient RNA helicase YTHDC2. Elife.

[43] Wu B., Li L., Huang Y., Ma J., Min J. (2017). Readers, writers and erasers of *N*(6)-methylated adenosine modification. Curr Opin Struct Biol.

[44] Zhao B.S., Roundtree I.A., He C. (2017). Post-transcriptional gene regulation by mRNA modifications. Nat Rev Mol Cell Biol.

[45] Li F., Zhao D., Wu J., Shi Y. (2014). Structure of the YTH domain of human YTHDF2 in complex with an m(6)A mononucleotide reveals an aromatic cage for m(6)A recognition. Cell Res.

[46] Xu C., Liu K., Ahmed H., Loppnau P., Schapira M., Min J. (2015). Structural basis for the discriminative recognition of *N*^6^-methyladenosine RNA by the human YT521-B homology domain family of proteins. J Biol Chem.

[47] Zhu T., Roundtree I.A., Wang P., Wang X., Wang L., Sun C. (2014). Crystal structure of the YTH domain of YTHDF2 reveals mechanism for recognition of *N*^6^-methyladenosine. Cell Res.

[48] Theler D., Dominguez C., Blatter M., Boudet J., Allain F.H. (2014). Solution structure of the YTH domain in complex with *N*^6^-methyladenosine RNA: a reader of methylated RNA. Nucleic Acids Res.

[49] Alarcon C.R., Goodarzi H., Lee H., Liu X., Tavazoie S., Tavazoie S.F. (2015). HNRNPA2B1 is a mediator of m(6)A-dependent nuclear RNA processing events. Cell.

[50] Chen K., Lu Z., Wang X., Fu Y., Luo G.Z., Liu N. (2015). High-resolution *N*(6) -methyladenosine (m(6) A) map using photo-crosslinking-assisted m(6)A sequencing. Angew Chem Int Ed Engl.

[51] Huang H., Weng H., Sun W., Qin X., Shi H., Wu H. (2018). Recognition of RNA *N*(6)-methyladenosine by IGF2BP proteins enhances mRNA stability and translation. Nat Cell Biol.

[52] Stoilov P., Rafalska I., Stamm S. (2002). YTH: a new domain in nuclear proteins. Trends Biochem Sci.

[53] Stoss O., Olbrich M., Hartmann A.M., Konig H., Memmott J., Andreadis A. (2001). The STAR/GSG family protein rSLM-2 regulates the selection of alternative splice sites. J Biol Chem.

[54] Harigaya Y., Tanaka H., Yamanaka S., Tanaka K., Watanabe Y., Tsutsumi C. (2006). Selective elimination of messenger RNA prevents an incidence of untimely meiosis. Nature.

[55] McPheeters D.S., Cremona N., Sunder S., Chen H.M., Averbeck N., Leatherwood J. (2009). A complex gene regulatory mechanism that operates at the nexus of multiple RNA processing decisions. Nat Struct Mol Biol.

[56] Yap K.L., Zhou M.M. (2011). Structure and mechanisms of lysine methylation recognition by the chromodomain in gene transcription. Biochemistry.

[57] Wang C., Zhu Y., Bao H., Jiang Y., Xu C., Wu J. (2016). A novel RNA-binding mode of the YTH domain reveals the mechanism for recognition of determinant of selective removal by Mmi1. Nucleic Acids Res.

[58] Wu B., Xu J., Su S., Liu H., Gan J., Ma J. (2017). Structural insights into the specific recognition of DSR by the YTH domain containing protein Mmi1. Biochem Biophys Res Commun.

[59] Meyer K.D., Patil D.P., Zhou J., Zinoviev A., Skabkin M.A., Elemento O. (2015). 5′ UTR m(6)A promotes cap-independent translation. Cell.

[60] Meyer K.D., Jaffrey S.R. (2017). Rethinking m(6)A readers, writers, and erasers. Annu Rev Cell Dev Biol.

[61] Wu B., Su S., Patil D.P., Liu H., Gan J., Jaffrey S.R. (2018). Molecular basis for the specific and multivariant recognitions of RNA substrates by human hnRNP A2/B1. Nat Commun.

[62] Nicastro G., Taylor I.A., Ramos A. (2015). KH-RNA interactions: back in the groove. Curr Opin Struct Biol.

[63] Phan A.T., Kuryavyi V., Darnell J.C., Serganov A., Majumdar A., Ilin S. (2011). Structure-function studies of FMRP RGG peptide recognition of an RNA duplex-quadruplex junction. Nat Struct Mol Biol.

[64] Patil D.P., Pickering B.F., Jaffrey S.R. (2018). Reading m(6)A in the transcriptome: m(6)A-binding proteins. Trends Cell Biol.

[65] Dominissini D., Nachtergaele S., Moshitch-Moshkovitz S., Peer E., Kol N., Ben-Haim M.S. (2016). The dynamic *N*(1)-methyladenosine methylome in eukaryotic messenger RNA. Nature.

[66] Yang X., Yang Y., Sun B.F., Chen Y.S., Xu J.W., Lai W.Y. (2017). 5-methylcytosine promotes mRNA export – NSUN2 as the methyltransferase and ALYREF as an m(5)C reader. Cell Res.

[67] Roundtree I.A., Evans M.E., Pan T., He C. (2017). Dynamic RNA modifications in gene expression regulation. Cell.

[68] Charette M., Gray M.W. (2000). Pseudouridine in RNA: what, where, how, and why. IUBMB Life.

[69] Arrowsmith C.H., Bountra C., Fish P.V., Lee K., Schapira M. (2012). Epigenetic protein families: a new frontier for drug discovery. Nat Rev Drug Discov.

[70] Cao F., Townsend E.C., Karatas H., Xu J., Li L., Lee S. (2014). Targeting MLL1 H3K4 methyltransferase activity in mixed-lineage leukemia. Mol Cell.

[71] Xu C., Bian C., Yang W., Galka M., Ouyang H., Chen C. (2010). Binding of different histone marks differentially regulates the activity and specificity of polycomb repressive complex 2 (PRC2). Proc Natl Acad Sci U S A.

